# *“How Can the Patients Remain Safe, If We Are Not Safe and Protected from the Infections”?* A Qualitative Exploration among Health-Care Workers about Challenges of Maintaining Hospital Cleanliness in a Resource Limited Tertiary Setting in Rural India

**DOI:** 10.3390/ijerph15091942

**Published:** 2018-09-06

**Authors:** Sudhir Chandra Joshi, Vishal Diwan, Rita Joshi, Megha Sharma, Ashish Pathak, Harshada Shah, Ashok J. Tamhankar, Cecilia Stålsby Lundborg

**Affiliations:** 1 Department of Community Medicine, R.D. Gardi Medical College, Ujjain 456006, India; sc.joshi@rdgmc.edu.in; 2 Department of Public Health and Environment, R.D. Gardi Medical College, Ujjain 456006, India; 3 International Centre for Health Research, Ujjain Charitable Trust Hospital and Research Centre, Ujjain 456001, India; 4 Department of Public Health Sciences, Global Health, Health Systems and Policy (HSP): Medicines Focusing Antibiotics, Karolinska Institutet, 17177 Stockholm, Sweden; megha.sharma@ki.se (M.S.); Ashish.Pathak@ki.se (A.P.); ashok.tamhankar@ki.se (A.J.T.); Cecilia.Stalsby.Lundborg@ki.se (C.S.T.); 5 Department of Microbiology, R.D. Gardi Medical College, Ujjain 456006, India; ritajoshi546@gmail.com (R.J.); h.shah@rdgmc.edu.in (H.S.); 6 Department of Pharmacology, R.D. Gardi Medical College, Ujjain 456006, India; 7 Department of Pediatrics, R.D. Gardi Medical College, Ujjain 456006, India; 8 Department of Women and Children’s Health, International Maternal and Child Health Unit, Uppsala University, 75185 Uppsala, Sweden; 9 Indian Initiative for Management of Antibiotic Resistance, Department of Environmental Medicine, R.D. Gardi Medical College, Ujjain 456006, India

**Keywords:** health care-associated infection, hospital environmental hygiene, infection control, patient safety, hospital personnel, qualitative research, India

## Abstract

*Background*: Health care-associated infections (HAIs) result in treatment delays as well as failures and financial losses not only to patients but also to the treating hospital and overall health-care delivery system. Due to hospital-acquired infections, there are problems of increase in morbidity and mortality, additional diagnostic and therapeutic interventions and ultimately antimicrobial resistance. Proper understanding among health-care workers about the ill effects of HAIs is very important to address this issue. The present study is a qualitative exploration aimed at understanding various aspects of hospital environmental hygiene and Infection prevention control program, by exploring the staff perception regarding the challenges, facilitators and barriers as well as feasible measures towards improvement in a rural tertiary teaching hospital in central India. *Method*: A qualitative study was conducted using 10 focus group discussions (FGDs) among five different professional groups, which included hospital administrators, doctors, nurses, environmental cleaning staff, and undergraduate medical students. The FGD guide included the following topics: (1) opinion about the status of cleanliness, (2) concepts and actual practices prevailing of hospital environmental hygiene, (3) Barriers, constraints, and problems in maintaining hospital environmental hygiene, (4) Suggestions for improvements. The data were analyzed manually using the content (thematic) analysis method. *Results*: Two themes were identified: Theme 1: “Prevailing practices and problems related to hospital surface/object contamination and hospital infection control”. Theme 2: “Measures suggested for improving hospital cleanliness within the existing constraints”. The participants emphasized the influence of resource constraints and needed inputs. They brought up the consequent prevailing practices and problems related, on one hand, to various stakeholders (service consumers, hospital personnel including the management), on the other, to specific infection prevention and control processes. They also suggested various measures for improvement. *Conclusions*: The study has revealed prevailing practices, problems, and suggested measures related to hospital environmental hygiene, particularly hospital cleanliness and HAI prevention and control processes. These insights and assertions are important for developing future behavioral and structural interventions in resource-limited settings. This study recommends a nationwide reliable HAI surveillance system and a robust infection prevention and control program in each health-care institution.

## 1. Background

Health care-associated infection (HAI) is an infection occurring in a patient during the process of care in a hospital or other health-care facility, which was not present or incubating at the time of admission. HAIs can also appear after discharge from the hospital and include occupational infections among hospital/health-care staff [1,2,3]. HAIs result in considerable human suffering and financial losses on account of prolonged hospital stays, increased morbidity and mortality, additional diagnostic and therapeutic interventions and antimicrobial resistance [4,5,6].

The burden of HAIs is disproportionately higher in low- and middle-income countries (LMICs) [2,7] as compared to high-income countries (HICs) [1,2].

In the hospital environment, patients contract HAIs through other patients or hospital staff; contaminated surfaces and instruments; dust; air; water; various objects (fomites) in the patient care areas [8,9,10,11,12]. Several factors such as lack of access to safe water, unclean instruments, and environmental surfaces, increased recourse to invasive medical devices, care of the critically ill, understaffing, high bed occupancy, and increased patient transfers all lead to the risk of HAIs [12,13,14]. Thus, hospital environmental hygiene has a very significant role in an Infection Prevention and Control Program as a part of “horizontal interventions” and should be integrated with hand hygiene and antimicrobial stewardship [10,15,16]. In the “vertical interventions” too, hospital environmental hygiene is crucial [9,15].

Increasing attention has recently been directed toward the importance of cleaning and cleanliness in HAIs prevention and control [4,9,10,11,17,18,19], which was neglected earlier [9,10,11,14,17,18,19]. Despite all these growing efforts, infection control in most developing countries remains either nonexistent or ineffective, posing a significant threat to quality of patient care [20,21,22]. International infection control guidelines have been established for improving infection prevention and control [23,24] but these can be implemented only when a level of basic infrastructure exists [25] and the structural interventions and changes are supported by the behavioral ones.

Surveillance systems for HAIs—a core part of an infection prevention and control program—exist in several HICs but are virtually nonexistent or too inadequate and unreliable in most LMICs, where, out of 147, only 23 countries have a functioning HAIs surveillance system [20]. Therefore, in the developing countries, HAI risk assessment from explorations on health-care workers’ perception, attitudes, behaviors, compliance, and/or perceived risks has become an alternative research strategy [26].

In India, the majority of health-care settings lack a robust infection prevention and control program and no nationwide HAIs surveillance system exists [23,27]. Nevertheless, as the scenario is changing at both the national and international levels, it is being anticipated that with the advance of antimicrobial resistance increasing for virtually all pathogens, the science underpinning infection prevention and control programs, including cleaning, will now attain a status hitherto unrecognized [9]. Research on these issues has been scanty, though only recently the scenario has changed [28,29].

Qualitative research methods are significant for gaining insights into infection prevention efforts. Infection control professionals and hospital epidemiologists have been accustomed to using quantitative methods. Qualitative methods are increasingly being used nowadays [30], for understanding ‘why’ and ‘how’ regarding various processes as well as in studying peoples’ perceptions. These are often used to explore concepts, feelings, opinions, interpretations, and meanings, or why people behave in a certain way [31]. Qualitative research methods are helpful in developing a deeper understanding of the factors influencing lives of patients and in implementing necessary changes [32].

In view of the foregoing, we are presenting here a qualitative exploration of the perception, experiences, and feelings of the administrative, clinical, and other staff categories including the cleaning staff of a charity-based, resource-limited, not-for-profit, nongovernmental, rural, tertiary care, teaching hospital in central India. The aim of this exploration is to contribute to understanding of the context of various aspects of hospital environmental hygiene and an infection prevention and control program, including environmental contamination, decontamination, and program-related issues, by exploring the perception regarding the challenges, facilitators, and barriers, as well as feasible measures towards improvement.

## 2. Material and Methods

The study was conducted in the tertiary rural teaching hospital in central India. The data were collected in conjunction with the data collection for previous papers, where details of participants, data collection, and data analyses have been presented [21,22]. Among 5 professional groups, 10 focus group discussions (FGDs) were conducted, 2 FGDs for each group. These included: hospital administrators, doctors, nursing staff, cleaning staff, and medical undergraduates (Table 1).

As the setting has hierarchical and traditional environments, the FGDs were conducted separately for various categories of participants, so that the staff in various hierarchical positions could express themselves freely. This also helped that the expressions of various staff categories remained confidential from each other. The subject of the FGDs was provided to the various categories of staff and they were invited to participate voluntarily. Written consent was obtained from each participant and they were informed that they had freedom to withdraw from the study at any time.

For data collection, the authors developed a preliminary topic guide after a review of the available literature and observations in the hospital. This was pretested by two pilot FGDs. On this basis, a final topic guide was developed and then the FGDs were conducted according to the language proficiency of the participants in English or Hindi or both, by S.C.J., R.J., H.S., and V.D.; one or two of them moderated the FGD as ‘facilitator(s)’ in rotation and the others worked as ‘observer/note-taker’. Discussion points that were included in FGDs were: (1) opinion about the status of cleanliness, (2) concepts and actual practices of hospital environmental hygiene, (3) barriers, constraints, and problems in maintaining hospital environmental hygiene, (4) suggestions for improvement. All the discussions were voice-recorded with the informed voluntary consent of the participants. The sound files were transcribed and the Hindi texts were all translated into English.

Data analysis of the texts was done manually. Latent and manifest content analysis method was applied [33], whereby ‘meaning units’ and ‘condensed meaning units’ were derived and ‘codes’ were extracted. Coding was performed by two authors independently (S.C.J. and R.J.). Codes were further grouped into various ‘categories’ out of which ‘subthemes’ and finally ‘themes’ were identified. Researcher triangulation was used to validate the findings and the results were repeatedly discussed among the authors who have varied backgrounds such as community medicine, clinical medicine, environmental medicine, medical microbiology, pharmacy and public health, have different genders, and come from India and Sweden. Due to ethical and legal restrictions, all inquiries should be made with The Chairman, Ethics Committee, R.D. Gardi Medical College, Agar Road, Ujjain, India 456006 (Emails: iecrdgmc@yahoo.in, uctharc@bsnl.in), giving all details of the publication. Upon verification of genuineness of the inquiry, the data will be made available. For reference, please quote ethical permission No. 169, dated 6 June 2011. Not applicable as we are not reporting individual participant’s data in any form.

## 3. Results

Two themes emerged in the process of thematic (content) analysis.

Theme 1: Prevailing practices and problems related to hospital surface/object contamination and hospital infection control.

Theme 2: Solutions suggested for improving hospital cleanliness within the existing constraints.

Theme 1 has emerged from four subthemes which are as follows: ‘related to the beneficiaries (service consumers)’, ‘related to hospital personnel and organizational work–culture’, ‘practices and problems related to the specific infection prevention and control processes’, and finally, ‘influence of resource constraints’.

Theme 2 is formed of two subthemes, ‘behavioral and structural interventions to address the prevailing practices and problems’ and ‘measures suggested for improving the specific hospital infection control processes’.

Both the themes are being described subtheme-wise as follows:

**Theme 1: Prevailing practices and problems related to hospital surface/object contamination and hospital infection control (Table 2).**

**Subtheme 1.1: Related to the beneficiaries (service consumers).**

**Categories:** Surface contamination by the beneficiaries/Beneficiary background/Visitor overcrowding.

Prevailing community practices and associated problems have surfaced in this subtheme. The study hospital being in a rural area, the patients bring in their customs and habits. A large number of visitors for each patient is a very common custom, sort of a ‘rule’ with rare exceptions, consequently the wards and even the intensive care units (ICUs) get overcrowded by visitors.

Besides visitor overcrowding, participants discussed rural background, poverty, powerlessness, illiteracy, ignorance, and diehard habits of the participants. Defecation on washroom floors and filth dissemination through spoiled shoe soles, spitting and littering anywhere or waste disposal through windows whereby the waste falls on the head of a person passing on the ground are some examples.

*“Mostly our patients they are from rural area. We are dealing with a large number of rural patients”.*

**(Administrators FGD 8)**

*“Mostly the filth spreads from the latrines and bathrooms. People are responsible for this they scatter the filth. And such a case that we go on cleaning and the filth always remains we clean daily but filth always remains there in spite of so much cleaning. Since floors have the filth, as people walk they spread the filth wherever they go.”*

**(Cleaning Staff FGD 6)**

**Subtheme 1.2: Related to the hospital personnel and organizational work culture.**

**Categories:** Surface contamination by the staff/Poor ward hygiene/Staff factors in visitor overcrowding/ Infection transmission in operation theatre (OT) /Infection Transmission in other critical zones.

This subtheme contains insights into the ways the hospital staff, including the doctors, substantially contribute to contamination of hospital floors and surfaces.

Doctors’ habit of throwing away the used contaminated needles on the floor and hospital administration’s and staff’s indifference to spillage of blood and body fluids on the floors are two examples. A senior nurse informed:

*“What is happening in the TB Ward? When a chest tube is put in place for removal of fluid then the fluid is drained out in any kind of a container. The relatives or visitors of the patients would accidentally spill the fluid all around on the ground. The floor is thus soiled. Staff shortage is there and sweepers are not available. Hence problem arises who would clean it?”*

**(Nursing Administrators FGD 10)**

Other examples are contamination through broom and mop as the broom spreads dust all over and the same mop is used repeatedly without any cleaning and disinfectants. An additional issue was the storage of the mop being used.

*“Mop is really very dirty and the water is seldom changed. Then the storage of the mop is another issue. She would store that mop in one of the toilets. And that toilet is no longer usable, there is nothing like a broom closet.”*

**(Doctors FGD 9)**

Poorly paid and improperly trained frequently changing personnel formed the main corpus of the paramedical staff. The language problem—very poor knowledge of the languages in which instructions are given—was also described as a huge challenge.

Participants also revealed various practices and problems linked to the immediate environment of the inpatients, mostly their beds and some attached objects such as lockers, bed linen, mattresses blankets, pillows, pillow covers, etc. Neglect of implementation of rules in general and that related to visitor control in particular also came up.

Infection transmission from wards to OT and flies in the OT were also a cause of concern.

*“Can....can you imagine flies in the O.T. sitting on the wound of the patients? What is that? Can you talk about that flies I don’t know about your OT I have seen umpteen times the flies have been sitting while operating, on either on the trolley operating trolley or on the wound.”*

**(Doctors FGD 9)**

Seniors’ noncompliance of standard guidelines for infection control in the OT and the prevailing practices of entering OT without changing or without slipper or sterile shoe covers were also narrated by the doctors. Clumping together of seriously ill infectious and immuno-compromised patients with mildly ill non communicable disease patients was yet another issue.

Several issues related to organizational work culture and attitudes, practices and problems regarding resource management have also emerged.

**Subtheme 1.3: Practices and problems related to the specific infection prevention and control processes.**

**Categories:** Sterilization of instruments/Disinfection /HAI-Surveillance/Resource-management issues.

In several FGDs, participants talked about use of unsterilized dressing trays for keeping sterilized instruments and re-use of instruments without sterilization particularly in cases of poor and rural patients.

*“Come to our dressing room and you will know. The tray, on which the dressing material has been presented to you, has not been sterilized for generations. [On] the same tray the new [autoclaved] material from the drum is poured on.”*

**(Doctors FGD 9)**

*“The doctors are often using the same instruments, used on one patient and they are frequently used on other patients without sterilizing. In fact, this is the major cause of infection, from patient to patient. If the patients is from well [to do] background then doctor get conscious about his cleanliness but not when the rural and poor patients are coming.”*

**(Medical Students FGD 5)**

The issues related to inadequate supply and consequent dilution of antiseptics and disinfectants were also discussed in great detail, along with several aspects of HAI surveillance. One among these was related to problems regarding the delivery of reports by the laboratory to the particular doctor who sent the swab.

**Subtheme 1.4: Influence of resource constraints.**

**Categories:** Water shortage/Material shortage/Staff shortage/Restricted access issues.

Shortage of funds and consequently that of staff as well as water and other facilities emerged as the crux. The most emphasized resource constraint was availability of water 24x7. Other pressing resource constraints were scarcity of gloves and masks and limited supply of antiseptics and disinfectants.

Staff shortage was perceived as a root of several such issues as high workload, consequent issues like workload on a few persons, someone always occupied with some task and many tasks at a time. This constraint was perceived to be problematic for both the staff as well as the patients. It was felt that the problem is further compounded by certain practices such as duty-roster lacunae that lead to further shortage of staff at night, non deployment of a night supervisor and a tradition of taking away helping hands without consulting or even informing. Besides dearth of separate and adequate nursing staff for units such as Neonatal Intensive Care Unit (NICU), perennial shortage of cleaning staff, vacancies of sanitary inspector, infection control officer, and infection control nurse also emerged as important prevailing problems.

Some participants believed that restricted access for beneficiaries to the hospital toilets had been forcing them to defecate in the open fields around and on the hospital (bathroom) floors. Similarly, the scarcity of hospital blankets had compelled the beneficiaries to bring in their own beddings and blankets.

One of the very senior nursing administrators said:

*“Toilets are not cleaned properly there are so many relatives are coming, bed occupancy also more, shortage of staff is there, water shortage is also there and all; like gloves, mask, caps that we are also not getting, means, we have to just wait for the items. So these all thing are there.”*

**(Nursing Administrators FGD 10)**

Members of cleaning staff raised issues related to their own protection from filth and infection, demanding gloves and other components of personal protection.

*“After all who has to clean when a patient spits or vomits on the floor? It is we; we have to do it with our bare hands. Today I cleaned the vomitus of a patient with bare hands before I came here. I collected that entire nasty thing in my palm with the other hand and in both the hands, like this (depicts through action too). Like this, like this I filled the vomitus in my flexed palm.”*

**(Cleaning staff FGD7)**

**Theme 2: Measures suggested for improving hospital cleanliness within the existing constraints (Table 3).**

**Subtheme 2.1: Behavioral and structural interventions to address the prevailing practices and problems.**

**Categories:** Educational measures/Motivational measures/ Team development/Monitoring and Supervision/Security-system and assistance/Teaching of nursing care and ward care/Resource management and maintenance.

The educational measures included awareness, inculcation, and training, while incentives were suggested as motivational measures, e.g., rise in salary and other material incentives, certificates, and competition. Nurses emphasized competition of ward cleanliness. Administrators differed widely in their views regarding monetary incentives. In each FGD, there was a clear emphasis on training as a solution to the problems and faults. A combination of awareness, training, and monitoring interventions was also suggested.

For patient-assistance and overcrowding-control, participants suggested that the security-system should be improved. According to some participants, the security guards should be instructed to keep watch, educate, and assist the patients and effectively carry out the visitor overcrowding management. Suggestions also included many commonly practiced methods of other settings which are not being properly implemented in the study area. One such suggestion was that the visiting hours must be fixed and the security guards should not allow unrestricted flow of the visitors, *whereas* the administrators suggested that the nursing staff should know the difference between the visitors and the attendants; the nursing administrators emphasized strict control on overcrowding.

Monitoring and supervision, teaching of nursing care and ward care, as well as resource management and maintenance were also discussed at length (Table 3). Measures for staff protection were also brought up, whereby it was emphasized that each staff member be protected by means of Hepatitis B immunization and various personal protection and barrier methods.

*“How can the patients remain safe, if we are not safe and protected from the infections?”*

**(Nurses FGD3)**

**Subtheme 2.2: Measures suggested for improving the specific hospital infection prevention and control processes.**

**Categories:** Improving surveillance/Improving isolation and ventilation/Improving decontamination.

For improving surveillance, participants suggested that days be fixed for sending the cultures from various points of the hospital and for distribution of the culture results to the sender. They also discussed various ways of improving this process.

*“We would like to send the swabs from the [points] where the nurses sit one thing and from each ward especially from the post-operative ward and postnatal ward.”*

**(Doctors FGD 9)**

*“Fumigation* *should be done with proper norms, at proper intervals.”*

**(Nursing Administrators FGD 10)**

Issues about other processes included proper decontamination, need for data on disinfectant use, improvisation of isolation and ventilation, and monitoring of all these processes (Table 3).

## 4. Discussion

Infection prevention and control programs of health-care institutions / hospitals involve several components. The most prominent among these are: hand hygiene, health-care waste management, and hospital environmental hygiene. Our previous studies in the similar settings have already reported perceptions regarding importance of and issues related to hand hygiene and health-care waste management [21,22].

The present study has explored challenges, facilitators, and barriers of the various aspects of implementation of hospital environmental hygiene and infection prevention and control program whereby prevailing practices, associated problems and possible solutions have been studied. The practices also included policies, provisions, and programs.

This exploration has revealed that both processes of hospital environmental hygiene—(1) reducing contamination and (2) its removal—happen to be grossly inadequate at various points. All stakeholders contributed to contamination in various ways, while its removal through implementation of various aspects of infection prevention and control program remained ignored. Hence deficiencies existed in all domains ranging from simple cleaning and prescribed precautions to disinfection and sterilization.

Several earlier studies conducted in the resource-limited institutions mostly belonging to the LMICs have reported practices similar to our findings [34,35,36,37,38], which are in sharp contrast to a study from a resource-rich institution in north India [27].

We found fairly satisfactory level of knowledge of hospital staff on one hand and the dismal scenario of practices revealed on the other hand. Similar to our results in a study from Karachi (Pakistan), ‘vast gap’ was found between the knowledge and practices of health-care personnel [34].

A study from Turkey has compared the situations and performances in the ‘reasonable resource’ context of the Netherlands with ‘limited resource’ constraints of their nation. Focusing on the contrast of the two contexts—rich and poor—the authors have enumerated major problems in the LMICs which are similar to our findings, such as deficient infrastructure of hospitals, low compliance, understaffing, overcrowding, heavy workload, problems related to personal protective equipment, delayed installation of infection control program [35]. This contrast can be further explained by another example. In our study, soiled bed sheets have been reported to be re-used for the next patient on account of scarcity of sheets compounded with laundry and logistics-related problems. In the Netherlands, each hospital bed is washed after patient discharge, prior to its use by the next patient, “like a car wash”. A centralized bed cleaning system is part of the Netherland’s four-pronged strategy [39].

The cleaning frequencies and mopping were also perceived as unsatisfactory. Improper and infrequent use of broom and mop, prevailing practice of mop storage after its use in toilets, and use of the same mop head for several days without cleaning it and without adding disinfectant in the bucket were discussed in detail by the participants. In a tertiary hospital in Delhi as well, “the mop was neither washed in laundry nor with hot water rinsed with a chlorine-releasing agent before storing it dry in the janitor for the night” [36]. According to their study, flexible cleaning frequencies are impractical, hospital policy for cleaning should be laid down very clearly and areas like OTs and ICUs should be cleaned more frequently [36].

We found the practice of use of equipment without proper sterilization. Earlier publications have also reported that even single-use medical devices are commonly reused, often inappropriately, in resource-limited settings because of cost constraints, giving rise to important legal and ethical questions [37]. Lack of sterilization capability has driven up health-care infection rates as well as limited access to health care, especially in low-resource environments [38].

Several deficiencies obstacles and barriers identified in our study are in fact the urgently needed inputs such as: adequate facilities, an on-going robust infection prevention and control program, sufficient number of properly trained and motivated staff and deployment of and delegation of authority to adequate and competent supervisors with technical and leadership capabilities. Participants in our study generally believed that if these are not provided, mere guidelines and rules cannot change the scenario. A similar qualitative study conducted in Mongolia has reported that policies guidelines and recommendations are not sufficient in assuring an effective implementation of infection control programmers. The authors have identified and classified barriers to formulation and implementation of infection control policy [20].

Solutions suggested in our study included improving and strengthening several aspects of infection prevention and control program such as organization and leadership, provisions and facilities; training and interdepartmental communication; overcrowding control and isolation; and special drives and audits for improving microbiological surveillance and supervision. A recent publication has also suggested similar solutions, e.g., training in conjunction with broader systems changes, including the establishment of functioning infection prevention and control program-related committees, implementing standard policies and protocols, and improving health management information systems to capture information on HAIs [40].

Through this exploration, we are able to pinpoint the areas which should not be ignored. Usually resource limitations and technical capability are the main perceived obstacles to the implementation of hospital standards [41]. Resources other than the material ones are essential, more easily available yet often ignored. A qualitative study on infection control practices conducted in Gujrat State of India—aimed at evaluation of an intervention for more effectiveness (within the constraints of limited resources)—has concluded that “less attention is paid to factors such as team relationship, leadership and problem-solving. In our study, measures like inculcation, motivation, team development, etc. have been emphatically suggested, highlighting that developing the leadership capability as well as the technical capability should be aimed at.

During the last two decades, in some high-income countries stringent measures have been introduced with good results. Some stakeholders perceived these measures as ‘target and terror regimes’ [42]. For institutions in the LMICs—where the consumer movements are feeble and need to be encouraged—the same cannot be exactly copied, yet regulations and stringent measures are equally needed while resources should be mobilized, be properly managed under a capable leadership, and a national program for HAIs prevention and control must be developed. A nation-wide reliable HAIs surveillance system should be built. A robust infection prevention and control program should come up at least in all nationally accredited health-care institutions through a well-funded, empowered, properly implemented and stringently monitored national program. Fortunately, realizing the threat of antimicrobial resistance, some encouraging efforts have recently come up in this direction in India [43].

## 5. Methodological Considerations

In this study, groupwise discussions were conducted with all categories of staff, ranging from administrators to cleaning staff, to understand wider perspective about hospital cleanliness. This comprehensiveness has not been very common in several previous published studies, where results of only one or two groups were presented. The authors of this study came from different educational and professional backgrounds. This gave strength in conducting discussion and later writing of the manuscript with different understandings of cleanliness as per their background. As authors who conducted this study belonged to the same hospital, there is a possibility that participants could not have put forth all their views freely. On the contrary, it is also possible that this might have led to a more open and free discussion. The finding of this study can be applicable to other such hospitals of similar settings and context.

## 6. Conclusions

The study has revealed prevailing practices, problems, and suggested measures related to hospital environmental hygiene, particularly hospital cleanliness, infection prevention and control processes and infection prevention and control programs. These insights are important for developing future behavioral and structural interventions. This study is particularly significant for understanding the context of and developing advocacy and other interventions for health-care institutions in resource-limited settings. Inclusion of the cleaning staff had not been done in the earlier studies and this too has made its findings very much relevant. We conclude and recommend that a nation-wide reliable HAI surveillance system and robust infection prevention and control programs in each health-care institution are urgently needed for which resources and other inputs should be mobilized and be properly managed.

## Figures and Tables

**Table 1:** Characteristics of participants.

	No.	Male	Female	Age Range (in Years)
Hospital Administrators	15	8	7	22–68
Doctors	15	11	4	26–63
Nursing Staff	14	0	14	20–34
Cleaning Staff	17	8	9	18–48
Undergraduate medical students	14	9	5	20–23
**Total**	**75**	**36**	**39**	**18–68**

**Table 2:** Theme 1—Prevailing practices and problems related to hospital surface/object contamination and hospital infection control.

Subtheme	Categories	Codes
1.1. Related to the beneficiaries (service consumers)	Surface contamination by the beneficiaries	defecation on washroom floors/spitting and littering anywhere/waste disposal through windows
	Beneficiary background	rural background/illiteracy/diehard habits
	Visitor overcrowding	tradition of overcrowding/sociocultural aspects/some doctors to justify
1.2. Related to hospital personnel and organizational work culture	Surface contamination by the staff	staff contributes significantly/spillage of blood and body fluids on the floors
	Poor ward hygiene	infrequent cleaning/linen and laundry/mattresses/blankets/pillows/pillow covers/inadequate patient care/lacunae in ward management
	Staff factors in visitor overcrowding	inadequate emphasis/treatment witness needed/need of some attenders/neglect of control by the security staff
	Infection transmission in OT *	flies in the OT/infection from ward to OT/seniors’ noncompliance in the OT
	Infection Transmission in other critical zones	infectious and immune compromised patients clumped together/casualty ward/ labor ward/ ICU **
1.3. Practices and problems related to the specific infection prevention and control processes	Sterilization of instruments	use of unsterilized instruments for poor and rural patients/use of unsterilized dressing trays and other similar lacunae/improper sterilization/re-use of unsterilized instruments
	Disinfection	dearth of disinfectants/dearth of antiseptics/excessive dilution/lacunae in procedures
	HAI-Surveillance	poor HAI surveillance/guideline ignorance/reporting problems
	Resource management issues	inculcation/competence and attitude related issues/lack of a proper system and infrastructure/pros and cons of the prevailing centralized system/poor maintenance of scarcely available facilities/lack of rules and regulations/poor clarity about rules/poor implementation of the rules/poor hygiene and sanitation/powerless hospital infection control committee/nominal security and control systems/lack of a team spirit/problems related to the management of cleaning staff
1.4. Influence of resource constraints	Water shortage	restricted water supply/wastage of scarce water/toilets stinking due to water shortage
	Material shortage	scarcity of PPE ^#^/availability of gloves and masks/ limited supply of antiseptics and disinfectants
	Staff shortage	staff shortage and high workload/duty roster lacunae and other deployment discrepancies/multitasking
	Restricted Access Issues	access to toilets/access to blankets

OT *: Operation Theatre; ICU **: Intensive Care Unit; HAI ^: Health care-associated infections; PPE ^#^: Personal Protective Equipment.

**Table 3:** Theme 2—Measures suggested for improving hospital cleanliness within the existing constraints.

Subtheme	Categories	Codes
2.1. Behavioral and structural interventions to address the prevailing practices and problems	Educational measures	awareness/inculcation/training/other avenues of improving competence/combination of educational measures with other interventions
	Motivational measures	rise in salary/other material incentives/certificates/competition and awards/positive-reinforcement/improving attitude
	Team development	Technical capability combined with leadership/team development
	Monitoring and Supervision	rules for everyone/implementation of rule/rounds/surprise-rounds/reinforcement-alternatives
	Security system and assistance	improving security system/visitor-overcrowding-management/patient-assistance/beneficiary-education-motivation
	Teaching of nursing care and ward care	improving nursing-care-teaching/improving bed-making/using the opportunity of bed-making/ ward-hygiene-teaching/personal-hygiene-care in wards
	Resource management and maintenance	adequate strength of staff/recruitment of ICO, ICN etc./provision of clean bed-linen/provision of blankets/provision of adequate number of washrooms and toilets/provision of water, mops, antiseptics and disinfectants/provisions of PPE for the staff and the visitors/standard precautions/universal precautions/hepatitis B immunization/barrier protection/availability of gloves and masks
2.2. Measures suggested for improving the specific hospital infection control processes	Improving surveillance	routine swabbing/delivery of reports/methods of surveillance/frequency and sites of surveillance/audit
	Improving isolation and ventilation	ventilation improvement priorities/isolation-improvisation/isolation-monitoring
	Improving decontamination	better methods for decontamination/proper decontamination/fumigation/need for data on disinfectant use

ICO: Infection Control Officer; ICN: Infection Control-Nurse; PPE: Personal Protective Equipment.
